# Impact of health insurance coverage on Chuna Manual Therapy utilization: A cross-sectional analysis using data from the 2017 and 2020 Korean Medicine Utilization and Herbal Medicine Consumption Survey

**DOI:** 10.1371/journal.pone.0321488

**Published:** 2025-05-08

**Authors:** Hyungsuk Kim, Chunhoo Cheon

**Affiliations:** 1 Department of Korean Medicine Rehabilitation, College of Korean Medicine, Kyung Hee University Korean Medicine Hospital, Kyung Hee University, Seoul, Republic of Korea; 2 Department of Preventive medicine, College of Korean Medicine, Kyung Hee University, Seoul, Republic of Korea; Cholistan University of Veterinary and Animal Sciences, PAKISTAN

## Abstract

Chuna Manual Therapy (CMT) is a component of traditional Korean Medicine that was integrated into national health insurance coverage of the Republic of Korea in 2019 to improve accessibility. This study investigates the impact of this policy change on CMT utilization patterns using data from the Korean Medicine Utilization and Herbal Medicine Consumption Survey, comparing pre-insurance (2017) and post-insurance (2020) periods. A descriptive cross-sectional analysis was conducted with a nationally representative sample, and logistic regression analysis was used to identify key determinants of CMT utilization. The results show a significant increase in CMT utilization, particularly among outpatients. Notable demographic shifts included an increased utilization among males, individuals with higher education levels, and those employed in professional, service, and skilled labor occupations. While insurance coverage improved accessibility, overall satisfaction with treatment results and costs slightly declined. Musculoskeletal disorders remained the primary conditions treated with CMT. The findings highlight that while insurance coverage expanded access to CMT, its effects on patient satisfaction and healthcare costs require further investigation. Further studies should assess the long-term implications of insurance coverage on both patient outcomes and healthcare provider practices.

## Introduction

Chuna Manual Therapy (CMT) is defined as articular mobilization or traction, or muscular relaxation or strengthening by using the hands or other body parts of Korean Medicine doctors (KMDs) [1]. CMT is one of the main therapeutic modalities within Korean Medicine, alongside acupuncture, moxibustion, and herbal medicine. The oldest evidence regarding the treatment method using hands of practitioners (manual therapy) can be found in a Thai artwork about 4,000 years ago, and CMT has evolved as of traditional Korean Medicine and by absorbing the concept and technique of Tuina, chiropractic, osteopathy, etc [2]. In the contemporary Korean Medicine era, this is one of the main treatment method along with acupuncture, moxibustion, herbal medicine, etc. It is usually used for fixing problems of musculoskeletal disorders by actually impact human structures and tissues such as bones, articulations, ligaments, tendons, muscles, nerves, and lymphatic system [3]. Recently, many researchers have been investigating and publishing articles regarding CMT for various conditions. The diseases covered in these researches are mainly musculoskeletal problems such as nonspecific chronic neck pain [4], temporomandibular disorders [5], whiplash injuries [6], but also neuropsychiatric and internal medicinal disorders like anorexia [7], and chronic obstructive pulmonary disease [8], respectively.

The Republic of Korea implements a National Health Insurance system, covering the majority of its citizens, while a subset of the low-income population benefits from medical aid [9]. Consequently, healthcare services under the insurance scheme are relatively affordable for the public. Calls for including CMT in the insurance system emerged as its utilization expanded in clinical practice [10]. In 2011, the Association of Korean Medicine officially announced its intention to advocate for the insurance coverage of CMT [10]. Following this, the Korean Society of Chuna Manual Medicine for Spine and Nerves conducted pivotal research studies in 2012 and 2014 to demonstrate the clinical effectiveness and feasibility of CMT insurance coverage [11,12].

In February 2015, the Health Insurance Policy Deliberation Committee under the Ministry of Health and Welfare included CMT insurance coverage in its mid-term plan, as part of broader efforts to enhance the accessibility of Korean Medicine [10]. This milestone led to the launch of a pilot program in February 2017, which 65 designated medical institutions, including 15 Korean Medicine hospitals and 50 Korean Medicine clinics, participating [13]. After the completion of the pilot program, CMT was fully integrated into the National Health Insurance system in 2019.

Under this policy, patients can access up to 20 sessions of CMT annually at reduced costs, with 50–80% of the treatment cost covered by insurance [2]. Simple CMT procedures, such as joint mobilizations and muscle relaxation techniques for musculoskeletal disorders, have a 50% copayment rate. Similarly, complex CMT procedures, including high-velocity low-amplitude techniques for conditions like disc problems and spinal stenosis, also have a 50% copayment rate. However, for complex CMT procedures addressing other musculoskeletal disorders, an 80% copayment rate is required [14].

Several studies have analyzed the effects of this insurance policy on CMT. For example, one study found that more patients with chronic conditions visited Korean Medicine hospitals after insurance coverage was implemented [15]. Another study concluded that regional factors significantly influenced CMT utilization before and after insurance coverage [16]. However, these studies primarily focused on the period following insurance coverage and lacked a comprehensive representation of all Korean Medicine users [17]. This study utilizes the Korean Medicine Utilization and Herbal Medicine Consumption Survey (KMUHMCS) data to compare the utilization patterns of CMT before (2017) and after (2020) the insurance coverage, providing a more inclusive evaluation of the impact of policy.

The KMUHMCS provides fundamental data for the formulating Korean Medicine policies by analyzing utilization patterns and behaviors related to Korean medical services [18]. Since health insurance coverage for CMT was implemented between the 4th (2017) and 5th (2020) surveys, this study compares data from these surveys to evaluate the impact of insurance coverage on CMT utilization patterns.

This study aims to understand the impact of health insurance coverage on the utilization of CMT by analyzing the data from the KMUHMCS surveys conducted in 2017 and 2020. Furthermore, it seeks to evaluate the broader effects of newly covered integrative and complementary medical technologies on healthcare utilization.

## Materials and methods

### Korean Medicine Utilization and Herbal Medicine Consumption Survey

This study was a descriptive cross-sectional analysis that utilized data from the KMUHMCS. The KMUHMCS produced fundamental data for the formulation of Korean Medicine policies and the development of the Korean Medicine industry by analyzing the utilization patterns, perceptions, and behaviors of the general public and users of outpatient and inpatient Korean medical services. The KMUHMCS was a comprehensive survey designed to analyze the utilization patterns of various Korean Medicine modalities, including herbal medicine and acupuncture. The 1st, 2nd, and 3rd surveys of the KMUHMCS were conducted in 2009, 2011, and 2014, respectively. However, microdata for these surveys were not publicly available, and only summary reports were released. This study utilized the 4th and 5th surveys, conducted in 2017 and 2020, respectively, as they provide publicly accessible microdata. Additionally, these surveys capture the periods before and after the implementation of health insurance coverage for CMT, which began in 2019. The 4th survey was conducted from September to December 2017, comprising face-to-face household interview with 5,000 members of the general public and interviews with 1,010 outpatients and 904 inpatients at medical institutions. The 5th survey was conducted from October 2020 to February 2021 using the same methodology, surveying 5,000 members of the general public, 1,000 outpatients, and 800 inpatients (600 from Korean medicine hospitals and clinics, and 200 from long-term care hospitals). This study specifically analyzes data from outpatients and inpatients who received CMT. The final analysis included 82 outpatients and 209 inpatients from 2017, and 200 outpatients and 254 inpatients from 2020. The study included aged 19 years or older who had utilized Korean Medicine services, with a specific focus on those who reported receiving CMT. Data collection was conducted through face-to-face exit interviews at medical institutions by trained professional interviewers. Participants completed a structured questionnaire via interviewer-administered or self-administered methods. The questionnaire encompassed sections on Korean Medicine service utilization patterns, perceptions of Korean Medicine, intention for future use and recommendations, health status, insurance coverage, public healthcare service experiences, and personal demographic information. This study followed the Strengthening the Reporting of Observational Studies in Epidemiology (STROBE) guidelines. The completed STROBE checklist is provided as Supporting Information (S1 Checklist).

### Variables

In this study, the following variables from the KMUHMCS were utilized: sex, age group, marital status, education level, type of occupation, household income, frequency and cost of Korean medical service utilization, region, primary symptoms or disease treated, perceived treatment effectiveness, satisfaction (treatment outcomes, treatment costs), perception of costs, private health insurance enrollment and coverage of Korean medical services in private insurance, intention for future use, and willingness to recommend to others. Quantitative variables were categorized based on the data provided. Age for outpatients was grouped into the following categories: under 40, 40–49, 50–59, and 60 years or older. For inpatients, age was categorized into 19–29, 30–39, 40–49, 50–59, and 60 years or older. Household income was divided into five groups: 1,500,000 KRW or less, 1,500,000–3,000,000 KRW, 3,000,000–4,500,000 KRW, 4,500,000–6,000,000 KRW, and more than 6,000,000 KRW. Medical expenses for outpatients were classified into the following categories: 10,000 KRW or less, 10,000–100,000 KRW, 100,000–500,000 KRW, 500,000–1,000,000 KRW, and over 1,000,000 KRW. For inpatients, the categories were 100,000 KRW or less, 100,000–500,000 KRW, 500,000–1,000,000 KRW, 1,000,000–2,000,000 KRW, 2,000,000–3,000,000 KRW, and over 3,000,000 KRW.

Perceived treatment effectiveness was assessed by asking participants how effective they thought the treatment was. Response included: not at all effective, not very effective, somewhat effective, very effective, and unknown. For analysis, responses of not at all effective and not very effective were grouped as “Not effective,” while “somewhat effective” and “very effective” were analyzed separately.

Satisfaction was assessed using the question, “How satisfied were you with the Korean Medicine services you used in the past year for each of the following items?” This included satisfaction with treatment results and satisfaction with treatment costs. Response included: very dissatisfied, slightly dissatisfied, neutral, slightly satisfied, and very satisfied. For analysis, slightly satisfied and very satisfied were grouped as “Satisfied,” while very dissatisfied, slightly dissatisfied, and neutral were grouped as “Below average.”

Future use intention was assessed by asking, “Do you intend to use Korean Medicine services if needed?” with response options of “Yes” or “No.” Willingness to recommend Korean Medicine services was assessed by asking, “Would you recommend Korean Medicine services to others?” with response options of “Yes” or “No.”

Perception of cost level was assessed by asking, “How would you rate the cost of utilizing Korean Medicine services?” Responses included: very expensive, slightly expensive, average, slightly inexpensive, and very inexpensive. For analysis, responses of very expensive and slightly expensive were grouped as “Expensive,” while average, slightly inexpensive, and very inexpensive were grouped as “Below average.”

Private health insurance enrollment was assessed by asking “Are you enrolled in private health insurance (e.g., disease or indemnity insurance)?” with response options of “Yes” or “No.” for those who answered “Yes,” a follow-up question asked whether their insurance included coverage for Korean Medicine services: “Does your private health insurance include coverage for Korean Medicine services?” Response included “Yes,” “No,” or “Unknown.”

### Statistical analysis

In this study, descriptive analyses were conducted using data from the 4th and 5th KMUHMCS. The KMUHMCS utilized a complex sampling design; therefore, analysis methods accounted for weight, strata and clusters to ensure representativeness of the overall Korean medicine user population. Descriptive statistics were used to summarize the demographic characteristics, utilization patterns, satisfaction, perceived effectiveness, and common diseases treated with CMT among outpatients and inpatients in 2017 and 2020. Absolute frequencies were presented as raw counts, while relative frequencies were reported considering the complex sampling design. To assess the factors associated with CMT utilization before and after the inclusion of CMT in national health insurance coverage, logistic regression analyses were conducted separately for outpatient and inpatient populations. The dependent variable was the utilization of CMT (0 = non-utilization, 1 = utilization). Independent variables included demographic and socioeconomic factors such as sex, age group, marital status, education level, occupational status, household income, region, type of institution, and private health insurance status. The results were expressed as odds ratios (ORs) with 95% confidence intervals (CIs). All analyses were performed using R version 4.3.1.

### Ethical statement

This study utilized secondary data from the KMUHMCS, a publicly available national statistical survey. The KMUHMCS data were fully anonymized prior to researcher access, ensuring no personally identifiable information was included. As such, this study did not involve direct interaction with participants, and the need for informed consent was waived. This research was reviewed and approved by the institutional review board of Kyung Hee University (registration number KHSIRB-24–491(EA)), which granted an exemption from ethical review based on the use of pre-existing, anonymized data.

## Results

### Demographic characteristics of outpatient CMT comparison between 2017 and 2020

Data from 2017 included 1,010 outpatients and 904 inpatients, and data from 2020 included 1,000 outpatients and 800 inpatients. No missing data were identified, and all eligible individuals were included in the final analysis. Among these, this study specifically focused on participants who received CMT. The final analysis included 82 outpatients from 2017, and 200 outpatients from 2020. The demographic characteristics of outpatient CMT patients exhibited several notable changes between 2017 and 2020 (Table 1). There was a significant increase in the proportion of male patients (from 39.5% in 2017 to 49.8% in 2020), suggesting a growing utilization among men. The age group of 50–59 years also saw a notable rise (20.5% to 28.9%). Educational attainment shifted markedly, with a higher proportion of patients having a college education or higher (28.7% to 57.3%), while those with only middle school education decreased (36.3% to 4.9%). Occupationally, there was an increase in service and sales workers (24.9% to 36.8%) and a decrease in homemakers (29.8% to 4.8%). Household income distribution showed a rise in higher-income patients (14.0% to 18.0%). Additionally, the frequency of CMT use per patient decreased, while expenditure patterns indicated a higher proportion of patients spending larger amounts. Regional distribution revealed a higher concentration of patients from the capital area (58.8% and 69.4%).

**Table 1:** Comparison of demographic characteristics of outpatient Chuna Manual Therapy patients between 2017 and 2020.

	2017		2020	
	n	%	n	%
Sex				
Male	37	39.5	92	49.8
Female	45	60.5	108	50.2
Age group				
≤ 40	19	33.0	69	27.0
40-49	20	22.1	35	21.2
50-59	19	20.5	52	28.9
≥ 60	24	24.4	44	22.9
Marital status				
Single	13	17.8	46	17.1
Married	69	82.2	154	82.9
Education level				
≤ Primary school	13	13.2	5	7.8
Middle school	30	36.3	13	4.9
High school	16	21.8	69	30.0
≥ College	23	28.7	113	57.3
Occupational status				
Professional/Managerial	10	8.3	29	13.9
Office/Clerical	14	18.5	59	29.2
Service/Sales	18	24.9	61	36.8
Agriculture/Fishing	7	9.5	3	0.3
Skilled Labor	2	1.8	19	10.0
Student	3	5.2	4	1.1
Homemaker	25	29.8	18	4.8
Unemployed	3	2.0	7	3.9
Household income (million Won)				
≤ 1.5 million Won	11	7.7	6	4.5
1.5 - 3.0	16	21.3	47	26.4
3.0 - 4.5	27	27.3	53	22.8
4.5 - 6.0	17	29.7	51	28.3
≥ 6.0 million Won	11	14.0	43	18.0
Frequency of use (mean ± SD)	9.5 ± 8.0		2.3 ± 0.9	
Expenditure (thousand Won)				
< 10	7	14.7	3	1.8
10 - 100	23	27.8	29	12.9
100 - 500	31	41.5	69	33.7
500 - 1000	14	13.8	54	30.7
≥ 1000	7	2.2	45	20.9
Region				
Capital area	44	58.8	137	69.4
Chungcheong province	6	10.3	21	11.7
Gyeongsang Province	3	10.7	15	10.6
Jeolla province	29	20.2	27	8.3
Type of institution				
Hospital	45	11.8	85	13.7
Clinic	37	88.2	115	86.3

**Fig 1:**
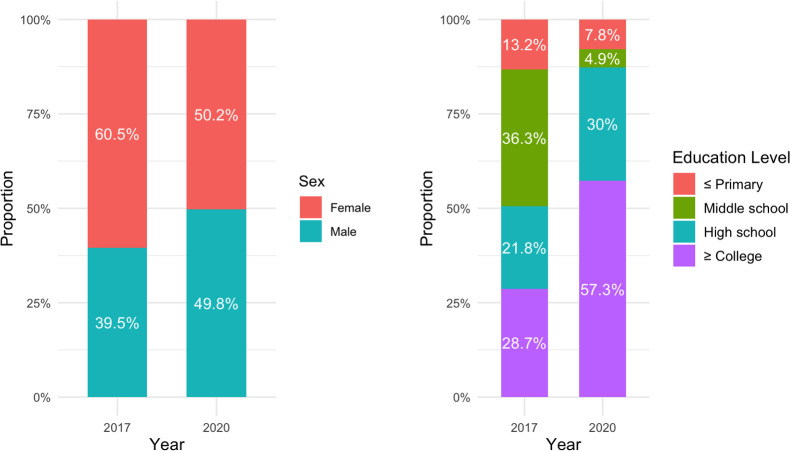
Sex distribution and educational attainment of outpatient CMT users in 2017 and 2020.

**Fig 2:**
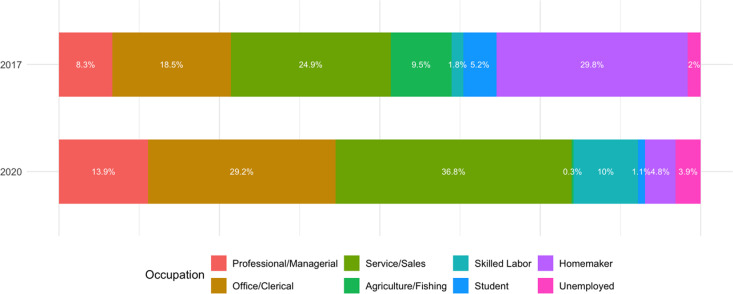
Occupational distribution of outpatient CMT users in 2017 and 2020.

To further illustrate these demographic changes, graphical representations of key aspects of CMT utilization have been included. Fig 1 shows the sex distribution and educational level of outpatient CMT users in 2017 and 2020, highlighting the increased proportion of male patients and the higher proportion of users with a high school diploma or higher in 2020. Additionally, Fig 2 visualizes the occupational distribution of CMT users, demonstrating the rise in service and sales workers and the decline in homemakers.

### Comparison of common disease treated with outpatient CMT in 2017 and 2020

In both 2017 and 2020, Lumbar Spine (Lower Back) issues were the most frequently treated symptoms with CMT, showing a significant increase from 36.2% in 2017 to 67.4% in 2020 (Table 2). In both 2017 and 2020, musculoskeletal disorders, including dislocations, sprains, and strains of various joints, as well as pain and disorders of the neck, shoulder, and back, were highly prevalent. Fracture and injury of the femur and lower leg, as well as rheumatoid arthritis, which were among the top 10 conditions in 2017, were not observed in 2020. Conversely, whiplash injury syndrome and migraine and other headaches emerged in the top 10 conditions in 2020 but were not present in 2017.

**Table 2:** Common disease treated with outpatient Chuna Manual Therapy.

	2017	n	%
1	Lumbar Spine (Lower Back)	35	36.2
2	Dislocation, Sprain, Strain, and Fracture of Lumbar and Pelvis	16	7.8
3	Arthritis and Joint Disorders (Knee, Elbow, Ankle, Wrist)	10	5.6
4	Dislocation, Sprain, and Strain of Joints (Shoulder, Knee, Wrist, Elbow, Ankle)	10	16.3
5	Cervical Spine (Neck)	6	5.5
6	Dislocation, Sprain, and Strain of Neck	6	14.9
7	Shoulder Disorders	5	3.8
8	Back Pain	4	3.4
9	Fracture and Injury of Femur and Lower Leg	3	12.5
10	Rheumatoid Arthritis	3	3.5
	2020	n	%
1	Lumbar Spine (Lower Back)	130	67.4
2	Cervical Spine (Neck)	33	14.5
3	Dislocation, Sprain, Strain, and Fracture of Lumbar and Pelvis	27	8.1
4	Arthritis and Joint Disorders (Knee, Elbow, Ankle, Wrist)	18	9.1
5	Dislocation, Sprain, and Strain of Joints (Shoulder, Knee, Wrist, Elbow, Ankle)	18	8.0
6	Shoulder Disorders	17	6.8
7	Back Pain	13	3.5
8	Dislocation, Sprain, and Strain of Neck	13	3.8
9	Whiplash Injury Syndrome	12	4.4
10	Migraine and Other Headaches	2	0.6

### Comparison of satisfaction and intention to use CMT among outpatients in 2017 and 2020

In comparing the satisfaction and intention to use Korean Medicine treatments between 2017 and 2020, several key trends were noted (Table 3). Overall satisfaction with treatment results and costs decreased in 2020 compared to 2017 (95.1% to 88.4% and 78.4% to 63.9% respectively). Despite these decreases, the perception of the effectiveness remained stable over the years. The perception of treatments being expensive increased (46.8% to 55.9%), but there was a notable rise in the number of patients with private health insurance coverage for Korean Medicine (37.4% to 65.9%). The intention to use Korean Medicine treatments in the future remained high, though it showed a slight decline. Additionally, the likelihood of recommending these treatments to others increased (88.0% to 92.7%).

**Table 3:** Comparison of outpatient satisfaction and intention to use between 2017 and 2020.

	2017		2020	
	n	%	n	%
Treatment effectiveness				
Very effective	47	59.4	96	58.7
Somewhat effective	33	40.3	56	40.3
Not effective	0	0	1	0.6
Unknown	1	0.3	2	0.4
Satisfaction with treatment results				
Satisfied	75	95.1	177	88.4
Below average	7	4.9	23	11.6
Satisfaction with treatment costs				
Satisfied	59	78.4	116	63.9
Below average	23	21.6	84	36.1
Perception of cost level				
Expensive	52	46.8	104	55.9
Below average	30	53.2	96	44.1
Private health insurance				
Yes	59	70.0	157	75.6
No	23	30.0	43	24.4
Inclusion of Korean Medicine in private insurance				
Yes	31	37.4	91	65.9
No	15	36.3	33	18.2
Not exactly	13	26.3	33	15.9
Future use intention				
Yes	77	98.8	191	94.6
No	5	1.2	9	5.4
Recommend to others				
Yes	71	88.0	186	92.7
No	11	12.0	14	7.3

### Demographic characteristics of inpatient CMT comparison between 2017 and 2020

For inpatients, the analysis was limited to individuals who received CMT, resulting in a final sample of 209 participants from 2017 and 254 participants from 2020. Between 2017 and 2020, the demographic characteristics of inpatient CMT patients revealed several notable trends (Table 4). The age distribution shifted, with a noticeable rise in patients aged 60 and above (18.4% to 31.9%). Educational attainment showed significant changes, with a higher proportion of patients having a college education or higher (27.7% to 42.6%), while those with only primary school education decreased markedly (19.8% to 2.3%). Occupationally, there was an increase in professional/managerial and service/sales workers, while the number of homemakers and agricultural/fishing workers decreased. Household income distribution showed mixed patterns without consistent directional changes. Expenditure patterns indicated a higher proportion of patients spending larger amounts. Regional distribution revealed an increase in patients from the Chungcheong province (4.3% to 10.2%) and a decrease from the Jeolla province (47.0% to 37.7%). Additionally, while hospitals remained the primary type of institution for inpatient CMT, there was an increase in the proportion of patients treated at clinics (7.8% to 19.5%).

**Table 4:** Comparison of demographic characteristics of inpatient Chuna Manual Therapy patients between 2017 and 2020.

	2017		2020	
	n	%	n	%
Sex				
Male	88	36.8	99	37.2
Female	121	63.2	155	62.8
Age group				
19-29	23	11.2	41	9.2
30-39	47	21.6	37	11.5
40-49	35	17.1	57	18.7
50-59	64	31.7	58	28.7
≥ 60	40	18.4	61	31.9
Marital status				
Single	44	21.0	73	19.2
Married	165	79.0	181	80.8
Education level				
≤ Primary school	39	19.8	6	2.3
Middle school	61	29.2	17	10.0
High school	49	23.3	104	45.1
≥ College	60	27.7	127	42.6
Occupational status				
Professional/Managerial	16	7.7	51	18.2
Office/Clerical	40	18.4	46	12.5
Service/Sales	40	18.8	54	21.4
Agriculture/Fishing	23	9.9	2	1.8
Skilled Labor	8	4.0	20	7.3
Student	10	4.6	4	0.7
Homemaker	65	33.2	51	27.5
Unemployed	7	3.4	26	10.6
Household income (million Won)				
≤ 1.5 million Won	9	4.5	28	14.2
1.5 - 3.0	56	26.6	80	33.8
3.0 - 4.5	70	33.2	63	22.7
4.5 - 6.0	50	24.4	38	14.8
≥ 6.0 million Won	24	11.3	45	14.5
Frequency of use (mean ± SD)	1.7 ± 1.2		1.4 ± 0.8	
Expenditure (thousand Won)				
< 100	20	8.8	27	8.2
100 -500	56	27.1	36	14.2
500 - 1000	57	27.6	54	20.4
1000 - 2000	35	16.4	53	24.3
2000–3000	17	8.1	30	13.8
≥ 3000	24	12.0	54	19.1
Region				
Capital area	92	46.9	149	45.9
Chungcheong province	11	4.3	37	10.2
Gyeongsang Province	5	1.8	17	6.2
Jeolla province	101	47.0	51	37.7
Type of institution				
Hospital	186	92.2	234	80.5
Clinic	23	7.8	20	19.5

### Comparison of common diseases treated with inpatient CMT in 2017 and 2020

Between 2017 and 2020, the common diseases treated with inpatient CMT revealed several notable trends (Table 5). Lumbar Spine (Lower Back) issues remained the most frequently treated condition in both years (49.2% and 44.5%). Cervical Spine (Neck) issues saw a significant increase in 2020 (14.2% to 32.5%). Dislocations, sprains, strains, and fractures of the lumbar and pelvis, as well as of various joints such as the shoulder, knee, wrist, elbow, and ankle, continued to be prevalent. New conditions like whiplash injury syndrome and migraines emerged as common issues in 2020, whereas conditions like injuries of the abdomen, lower back and thorax, as well as thoracic dislocations and strains, which were among the top conditions in 2017, were no longer observed.

**Table 5:** Common disease treated with inpatient Chuna Manual Therapy.

	2017	n	%
1	Lumbar Spine (Lower Back)	102	49.2
2	Dislocation, Sprain, and Strain of Joints (Shoulder, Knee, Wrist, Elbow, Ankle)	43	20.3
3	Dislocation, Sprain, Strain, and Fracture of Lumbar and Pelvis	42	20.5
4	Cervical Spine (Neck)	29	14.2
5	Arthritis and Joint Disorders (Knee, Elbow, Ankle, Wrist)	25	11.8
6	Dislocation, Sprain, and Strain of Neck	15	7.6
7	Shoulder Disorders	15	7.5
8	Injuries of the abdomen, lower back and pelvis	8	4.1
9	Back Pain	7	3.7
10	Dislocation, Sprain, and Strain of thorax	3	1.5
	2020	n	%
1	Lumbar Spine (Lower Back)	124	44.5
2	Cervical Spine (Neck)	87	32.5
3	Dislocation, Sprain, Strain, and Fracture of Lumbar and Pelvis	64	28.2
4	Dislocation, Sprain, and Strain of Joints (Shoulder, Knee, Wrist, Elbow, Ankle)	69	27.9
5	Shoulder Disorders	52	24.6
6	Dislocation, Sprain, and Strain of Neck	52	23.2
7	Arthritis and Joint Disorders (Knee, Elbow, Ankle, Wrist)	37	15.4
8	Back Pain	28	10.1
9	Whiplash Injury Syndrome	17	7.6
10	Migraine and Other Headaches	14	4.5

### Comparison of satisfaction and intention to use CMT among inpatients in 2017 and 2020

Between 2017 and 2020, the satisfaction and intention to use CMT among inpatients exhibited several notable trends (Table 6). Overall, the perception of treatment effectiveness improved, with a higher proportion of patients rating the treatment as very effective in 2020 compared to 2017 (from 66.0% in 2017 to 72.7% in 2020). However, there was a slight decrease in overall satisfaction with treatment results and costs in 2020 (93.6% to 86.0% and 67.4% to 59.7%, respectively). Perception of the cost level of treatments shifted, with fewer patients considering the treatments expensive in 2020 (76.5% to 54.0%). Regarding insurance, the inclusion of Korean Medicine in private insurance showed a notable decrease. Fewer patients reported that Korean Medicine was included in their private insurance plans in 2020 compared to 2017 (74.5% to 49.9%). The intention to use Korean Medicine in the future remained very high and even slightly increased (94.1% to 96.7%). Additionally, the likelihood of recommending CMT to others also showed a positive trend (92.6% to 97.3%).

**Table 6:** Comparison of inpatient satisfaction and intention to use between 2017 and 2020.

	2017		2020	
	n	%	n	%
Treatment effectiveness				
Very effective	137	66.0	176	72.7
Somewhat effective	63	29.5	71	24.3
Not effective	5	2.5	5	1.8
Unknown	4	2.1	2	1.2
Satisfaction with treatment results				
Satisfied	195	93.6	222	86.0
Below average	14	6.4	32	14.0
Satisfaction with treatment costs				
Satisfied	142	67.4	145	59.7
Below average	67	32.6	109	40.3
Perception of cost level				
Expensive	159	76.5	129	54.0
Below average	50	23.5	125	46.0
Private health insurance				
Yes	180	86.3	230	89.2
No	29	13.7	24	10.8
Inclusion of Korean Medicine in private insurance				
Yes	133	74.5	109	49.9
No	16	8.8	39	18.3
Not exactly	31	16.7	82	31.8
Future use intention				
Yes	197	94.1	244	96.7
No	12	5.9	10	3.3
Recommend to others				
Yes	192	92.6	240	97.3
No	17	7.4	14	2.7

### Logistic regression analysis on the determinants of CMT utilization among outpatients

The results of the logistic regression analysis for outpatients are summarized in Table 7. In the pre-insurance period (2017), no significant associations were found between CMT utilization and any of the examined variables among all Korean medicine outpatients. However, in the post-insurance period (2020), occupational status emerged as a significant factor influencing CMT utilization. Compared to homemakers, individuals in various occupational categories, including professional/managerial (OR = 10.25, 95% CI: 2.78–37.73), office/clerical (OR = 10.42, 95% CI: 3.38–32.13), service/sales (OR = 7.02, 95% CI: 2.33–21.14), and skilled labor (OR = 6.17, 95% CI: 1.44–26.36), exhibited significantly higher odds of receiving CMT. Regional disparities in CMT utilization were also observed, with patients in the capital area having significantly higher odds of receiving CMT compared to those in other regions. Specifically, patients in Gyeongsang Province (OR = 0.23, 95% CI: 0.09–0.54) and Jeolla Province (OR = 0.33, 95% CI: 0.12–0.92) exhibited lower likelihoods of CMT utilization relative to those in the capital area. Furthermore, the type of institution played a significant role, with patients receiving care at hospitals exhibiting higher odds of utilizing CMT compared to those treated at clinics (OR = 0.23, 95% CI: 0.13–0.40).

**Table 7:** Determinants of CMT utilization among outpatients.

	2017		2020	
	OR	CI	OR	CI
Sex				
Male	–		–	
Female	0.92	0.37-2.29	0.97	0.56-1.71
Age group				
≤ 40	–		–	
40-49	1.12	0.33-3.79	1.06	0.54-2.09
50-59	0.91	0.22-3.81	1.58	0.73-3.43
≥ 60	1.05	0.13-8.55	1.00	0.43-2.35
Marital status				
Single	–		–	
Married	0.67	0.15-2.96	1.58	0.76-3.32
Education level				
≤ Primary school	–		–	
Middle school	1.31	0.62-2.77	0.48	0.12-1.89
High school	1.66	0.52-5.27	0.59	0.13-2.62
≥ College	1.25	0.30-5.15	1.09	0.23-5.24
Occupational status				
Professional/Managerial	1.36	0.31-6.00	10.25	2.78-37.73
Office/Clerical	0.52	0.14-1.95	10.42	3.38-32.13
Service/Sales	0.52	0.19-1.38	7.02	2.33-21.14
Agriculture/Fishing	0.91	0.28-3.01	0.50	0.07-3.34
Skilled Labor	0.73	0.08-6.43	6.17	1.44-26.36
Student	1.03	0.08-12.71	3.54	0.47-26.7
Homemaker	–		–	
Unemployed	0.25	0.04-1.78	2.77	0.91-8.45
Household income (million Won)				
≤ 1.5 million Won	–		–	
1.5 - 3.0	0.75	0.22-2.57	0.44	0.09-2.19
3.0 - 4.5	0.92	0.29-2.94	0.22	0.04-1.14
4.5 - 6.0	1.96	0.57-6.69	0.26	0.05-1.49
≥ 6.0 million Won	0.88	0.19-4.22	0.40	0.08-1.92
Region				
Capital area	–		–	
Chungcheong province	0.72	0.23-2.27	0.51	0.18-1.42
Gyeongsang Province	0.32	0.07-1.43	0.23	0.09-0.54
Jeolla province	1.92	0.33-11.37	0.33	0.12-0.92
Type of institution				
Hospital	–		–	
Clinic	0.55	0.15-2.00	0.23	0.13-0.40
Private health insurance				
Yes	–		–	
No	0.94	0.32-2.78	0.62	0.24-1.58

### Logistic regression analysis on the determinants of CMT utilization among inpatients

Table 8 summarizes the logistic regression results for inpatients. In the pre-insurance period (2017), older age was associated with a higher likelihood of CMT utilization, with patients aged 50–59 exhibiting significantly greater odds (OR = 2.61, 95% CI: 1.08–6.33) compared to those aged 19–29. However, in the post-insurance period (2020), this trend reversed, with younger patients (19–29 years) being more likely to utilize CMT. Regarding education level, in 2017, patients with a middle school education had significantly lower odds of CMT utilization compared to those with primary school education or lower, but this difference was no longer significant in 2020. Occupational status also showed notable changes: in 2017, unemployed individuals had significantly lower odds of CMT utilization compared to homemakers, whereas in 2020, patients in the skilled labor (OR = 0.36, 95% CI: 0.15–0.88) and student (OR = 0.07, 95% CI: 0.01–0.36) groups were significantly less likely to receive CMT. Household income was a strong determinant in 2017, with higher-income groups exhibiting significantly greater odds of CMT utilization compared to those earning ≤1.5 million Won. However, in 2020, these income-related differences disappeared. Regional disparities were observed in both time periods, with CMT utilization being significantly higher in the capital area compared to Gyeongsang Province in 2017 and to both Gyeongsang and Jeolla Provinces in 2020. Unlike in outpatient settings, where hospital-based care was more associated with CMT, in the inpatient setting, Korean medicine clinics exhibited significantly higher odds of CMT utilization compared to hospitals (OR = 2.61, 95% CI: 1.13–6.04 in 2020).

**Table 8:** Determinants of CMT utilization among inpatients.

	2017		2020	
	OR	CI	OR	CI
Sex				
Male	–		–	
Female	0.76	0.49-1.18	0.68	0.41-1.12
Age group				
19-29	–			
30-39	2.21	0.97-5.04	0.36	0.15-0.86
40-49	1.87	0.77-4.54	0.40	0.16-0.98
50-59	2.61	1.08-6.33	0.32	0.12-0.81
≥ 60	1.79	0.61-5.28	0.21	0.07-0.60
Marital status				
Single	–			
Married	0.53	0.28-1.00	1.46	0.71-2.98
Education level				
≤ Primary school	–			
Middle school	0.37	0.20-0.66	2.20	0.61-7.94
High school	0.71	0.34-1.48	1.55	0.48-5.04
≥ College	0.73	0.34-1.56	1.09	0.32-3.68
Occupational status				
Professional/Managerial	0.59	0.29-1.18	0.85	0.43-1.71
Office/Clerical	0.57	0.29-1.12	1.01	0.39-2.60
Service/Sales	0.60	0.33-1.08	0.73	0.38-1.38
Agriculture/Fishing	0.87	0.41-1.85	0.72	0.14-3.61
Skilled Labor	1.59	0.58-4.35	0.36	0.15-0.88
Student	1.14	0.34-3.88	0.07	0.01-0.36
Homemaker	–			
Unemployed	0.33	0.15-0.70	0.61	0.28-1.35
Household income (million Won)				
≤ 1.5 million Won	–			
1.5 - 3.0	3.27	1.65-6.48	0.99	0.50-1.98
3.0 - 4.5	3.87	1.84-8.15	0.70	0.35-1.42
4.5 - 6.0	5.60	2.35-13.30	0.61	0.29-1.31
≥ 6.0 million Won	4.69	1.73-12.72	0.70	0.31-1.59
Region				
Capital area	–			
Chungcheong province	0.80	0.15-4.30	1.82	0.91-3.66
Gyeongsang Province	0.20	0.07-0.56	0.30	0.14-0.66
Jeolla province	0.97	0.53-1.77	0.35	0.22-0.56
Type of institution				
Hospital	–			
Clinic	1.10	0.54-2.24	2.61	1.13-6.04
Private health insurance				
Yes	–			
No	0.98	0.57-1.70	0.93	0.46-1.90

## Discussion

The most significant change observed following the application of health insurance to CMT was an increase in the number of patients receiving the treatment. This increase appears to be due to enhanced patient accessibility resulting from reduced medical costs and more KMDs offering CMT following its inclusion under health insurance coverage [15]. Another notable shift was observed in the demographic characteristics of outpatient clients. Previous studies have indicated that the use of Korean Medicine was predominantly among women, the elderly, and individuals with lower educational levels [19]. Similarly, prior to insurance coverage, CMT usage followed this trend. However, post-insurance data reveals a notable shift in the demographic composition of CMT users, with a relative increase in the proportion of male patients, individuals with higher educational backgrounds, and those employed in office, service, and skilled labor sectors. This finding contrasts with previous research indicating that healthcare services in South Korea are generally utilized more frequently by female patients [20]. Additionally, prior studies have suggested that individuals with lower socioeconomic status—including lower educational attainment and income levels—tend to experience greater improvements in healthcare accessibility following health insurance expansion [21]. Given that educational attainment and occupation are key indicators of socioeconomic status, the observed trend suggests that the increased utilization of CMT may be more attributable to the characteristics of CMT itself rather than the expansion of insurance coverage. These demographic shifts can be explained by two main factors: first, the conditions necessitating CMT that are covered by health insurance occur more frequently among men and those in office, service, and skilled labor professions. Second, the promotion of insured CMT was extensively conducted on social media, which is more readily accessed by younger, highly educated individuals. These factors likely contributed to the increased utilization of CMT among these specific demographic groups.

Moreover, the proportion of patients incurring higher expenditures on Korean Medicine has increased. This increase in expenditure is not so much a reflection of higher costs associated with CMT itself, as health insurance may have actually reduced the direct costs of CMT. Rather, this suggests that patients receiving CMT are likely undergoing additional treatments, contributing to the overall increase in spending categories. This trend aligns with previous studies suggesting that the insurance coverage of CMT has been associated with an increase in other Korean Medicine treatments, such as physiotherapy, at Korean Medicine institutions [16].

The use of CMT also increased in the capital region while decreasing in the Jeolla region. This trend may be attributed to an increase in the number of Korean Medicine hospitals in the capital area and a decline in the Jeolla region [22].

The increase in inpatient usage was not as significant as that observed in outpatient settings, and changes were relatively minimal. This could be attributed to a high proportion of patients already receiving coverage for treatment costs through private insurance at Korean Medicine institutions, which likely lessened the impact of health insurance implementation on inpatient services compared to outpatient services. The fact that a high percentage of inpatients reported in 2017 that Korean Medicine was covered by private insurance supports this assumption. Previous studies have also indicated that individuals with private health insurance are more likely to utilize inpatient services [23,24]. The patterns of change were similar to those observed in outpatient services, and there was an increase in the proportion of inpatients at clinics offering Korean Medicine. This increase is more likely related to the growth in clinics equipped with inpatient facilities during this period than to the application of health insurance to CMT [25].

In the context of outpatient treatment, there were no significant shifts in the primary symptoms treated between 2017 and 2020, although the proportion of patients receiving care for lower back issues increased. Similarly, for inpatient settings, the primary symptoms treated remained largely consistent between these two years. However, there was a notable rise in the number of patients treated for conditions related to traffic accidents and headaches in 2020. The application of health insurance coverage did not appear to significantly alter the primary symptoms treated for both outpatient and inpatient care, likely because the conditions already commonly treated, predominantly musculoskeletal disorders, were those included under the insurance coverage. This is in line with a previous report that musculoskeletal conditions, including spinal disorders such as spinal stenosis and other back-related conditions, constituted the largest portion of hospitalized patients receiving CMT, accounting for 65% [16]. Expanding health insurance to cover additional indications, such as internal diseases, may lead to a broader range of conditions treated with CMT, potentially altering patient demographics.

Following the introduction of insurance coverage for CMT in 2019, the increase in the number of patients receiving CMT can be partially attributed to more KMDs offering the treatment in their clinics [26]. Previously, CMT was predominantly performed by a relatively small number of practitioners specifically interested in manual therapy. Unlike medications administered orally or via injection, intervention such as CMT that involve manual technique and diagnostics may be partially influenced by the level of skill of practitioner [27]. Based solely on this study, it is difficult to state definitively that the decreased satisfaction observed in post-insurance coverage survey is due to practitioners inexperienced with CMT. Additionally, the introduction of CMT under insurance coverage may have prompted individuals who are generally less favorable towards Korean Medicine to undergo CMT treatments. Further research is necessary to explore these possibilities more thoroughly.

In logistic regression, the results suggest that the policy change contributed to broadening the demographic profile of CMT users. Notably, CMT utilization increased among men and individuals in various occupational groups, including professionals, office workers, service industry employees, and skilled laborers. This indicates that the policy may have facilitated access for individuals with different work environments and healthcare needs. This shift could also be partially attributed to promotional strategies that primarily utilized digital platforms, such as social media and YouTube, which may have influenced a broad range of individuals [28]. In terms of regional differences, the accessibility improvements were more pronounced in the capital area, suggesting that regional disparities in healthcare infrastructure may have influenced the impact of the policy [29]. Institutional differences were also observed, with CMT utilization being higher in Korean Medicine hospitals for outpatients, while clinics were more frequently used for inpatient care. This pattern may be influenced by the increasing number of clinics offering inpatient facilities [18]. While accessibility improvements are generally expected with insurance coverage [30], in our study, this effect was observed only among inpatients.

Our research provides a broad and comprehensive analysis of the effects of health insurance coverage on CMT, contrasting with previous studies that focused more narrowly or on specific aspects. Previous research on the utilization patterns post-insurance implementation provides valuable insights but lack comparisons with pre-implementation data, missing a key aspect of assessing overall trends and impacts. For instance, one study analyzed the distribution and usage rates of various CMT practices within medical institutions from 2019 to 2021 [17], while another investigated patient demographics and therapy uptake during a specific two-month period at one hospital, primarily focusing on the immediate effects of insurance coverage on patient behavior [15]. In contrast, our research utilizes data from the KMUHMCS that spans both before and after the implementation of health insurance coverage. This approach allows us to conduct a detailed before-and-after comparison, providing a comprehensive analysis that captures the broader effects of health insurance on CMT utilization. Moreover, another study, while employing a sophisticated statistical model to analyze changes in CMT use and non-reimbursable treatment, relied on data from a large-scale survey of medical institutions, attempting to represent national trends [16]. However, this study did not utilize official national statistics and primarily focused on variations in usage patterns and changes in non-reimbursed services. Our research builds on these findings by also examining dimensions such as patient satisfaction and changes in the primary symptoms treated with CMT. By incorporating these additional factors, our study provides a more comprehensive view of how health insurance coverage influences not only the utilization rates but also the overall acceptance and affordability of CMT within the context of Korean Medicine.

### Limitations

The primary limitation of this study lies in its sampling design, which includes only a small subset of patients who have received CMT. This limitation makes it challenging to draw robust inferences regarding detailed aspects of CMT usage. Nevertheless, the data source remains the only available national statistic that represents the entirety of Korean Medicine users. It is also the sole dataset that includes information on CMT usage before and after the implementation of health insurance coverage, which enhances the significance of the findings. Another important limitation is the policy context in which this study was conducted. The inclusion of CMT—a widely practiced therapeutic modality—within the national health insurance system represents an unprecedented policy change in Korean Medicine [31]. Since there are only a limited number of comparable cases both domestically and internationally, interpreting our findings within a broader healthcare policy framework remains challenging. This lack of directly relevant literature has further complicated the interpretation of our results and restricted the ability to draw meaningful comparisons. To address these limitations, future research should employ a specialized survey designed specifically to investigate CMT in greater depth. This survey should include detailed questions regarding the duration and frequency of CMT treatments, as well as any adverse event following treatment. By exploring these aspects, we can improve our understanding of effectiveness of and broader implications of CMT in real-world settings.

## Conclusion

In conclusion, this study provides clear evidence of the impact of health insurance coverage on the utilization and accessibility of CMT. Our findings indicate a significant increase in the number of patients receiving CMT, particularly in outpatient settings. Given that health insurance coverage was mainly applied to musculoskeletal disorders, which were already the primary conditions treated with CMT, there were no notable shifts in the primary symptoms treated. However, the observed decline in patient satisfaction regarding treatment results and costs highlights the necessity for continuous research. Further studies are essential to comprehensively evaluate the long-term effects of insurance coverage on CMT utilization and patient outcomes.

## Supporting information

S1 Checklist STROBE checklist for cross-sectional studies. (PDF)
